# Association between tobacco use and body mass index in urban Indian population: implications for public health in India

**DOI:** 10.1186/1471-2458-6-70

**Published:** 2006-03-16

**Authors:** Mangesh S Pednekar, Prakash C Gupta, Heema C Shukla, James R Hebert

**Affiliations:** 1 Healis Sekhsaria Institute for Public Health, 601, Great Eastern Chambers, Plot No 28, Sector 11, CBD Belapur, Navi-Mumbai 400614, India; 2 Directorate of Public Health & Health Improvement, Hillingdon PCT, Kirk House, 97-109 High Street, Yiewsley, West Drayton, Middlesex UB7 7HJ, UK; 3 Department of Epidemiology and Biostatistics, Arnold School of Public health, University of South Carolina, 800 Sumter Street, Columbia, SC 29203, USA

## Abstract

**Background:**

Body mass index [BMI, weight (kg)/height (m^2^)], a measure of relative weight, is a good overall indicator of nutritional status and predictor of overall health. As in many developing countries, the high prevalence of very low BMIs in India represents an important public health risk. Tobacco, smoked in the form of cigarettes or bidis (handmade by rolling a dried rectangular piece of temburni leaf with 0.15–0.25 g of tobacco) or chewed, is another important determinant of health. Tobacco use also may exert a strong influence on BMI.

**Methods:**

The relationship between very low BMI (< 18.5 kg/m^2^) and tobacco use was examined using data from a representative cross-sectional survey of 99,598 adults (40,071 men and 59,527 women) carried out in the city of Mumbai (formerly known as Bombay) in western India. Participants were men and women aged ≥ 35 years who were residents of the main city of Mumbai.

**Results:**

All forms of tobacco use were associated with low BMI. The prevalence of low BMI was highest in bidi-smokers (32% compared to 13% in non-users). For smokers, the adjusted odds ratio (OR) and 95% confidence interval (CI) were OR = 1.80(1.65 to 1.96) for men and OR = 1.59(1.09 to 2.32) for women, respectively, relative to non-users. For smokeless tobacco and mixed habits (smoking and smokeless tobacco), OR = 1.28(1.19 to 1.38) and OR = 1.83(1.67 to 2.00) for men and OR = 1.50(1.43 to 1.59) and OR = 2.19(1.90 to 3.41) for women, respectively.

**Conclusion:**

Tobacco use appears to be an independent risk factor for low BMI in this population. We conclude that in such populations tobacco control research and interventions will need to be conducted in concert with nutrition research and interventions in order to improve the overall health status of the population.

## Background

Body mass index [BMI, weight (kg)/height (m^2^)], a measure of weight adjusted for height, is a simple and inexpensive index that is often used as a proxy for overall health of populations. [1] Body habitus, as described by BMI, is related to skeletal size, muscle mass, and adiposity. As such, it is influenced by diet, other aspects of lifestyle, and other environmental factors. The association between low BMI and smoking is well documented. [2,3] However, recent studies show that the nature of this relationship depends on educational level, gender, ethnicity, and frequency (e.g., number of cigarettes/day) of smoking. Often, the relationship changes with time. In Finland, for example, the inverse association between smoking and BMI weakened between 1982–1987 and became positive later. [4] Educational attainment is known to influence the relation between smoking and BMI in North European populations. [5,6] In these populations, smoking is inversely associated with BMI at lower levels of education and positively associated with BMI at higher levels of education. A U-shaped relation between frequency of smoking and BMI has been observed by some investigators. [7]

In India, tobacco is used in various forms. [8] In addition to smoking cigarettes, bidis are commonly smoked, as they are much cheaper. Bidis are handmade by rolling a dried rectangular piece of temburni leaf (*Diospyros melanoxylon*) with 0.15–0.25 g of sun-dried, flaxed tobacco. In addition, use of smokeless tobacco, in a variety of forms, is widespread among both men and women. The most common form of smokeless tobacco use is mishri, a black powder obtained by roasting and powdering tobacco, which is then applied to the gums using a finger. Another common form is chewing of betel-quid, a combination of betel-leaf, areca nut, slaked lime, tobacco, and condiments; combinations of ingredients are altered according to individual preferences. Use of all forms of tobacco is associated with higher all-cause mortality in the Indian population. [9-11] Previously, we reported that tobacco using (smoking and smokeless) is associated with low BMI in an Indian population. [12] The high prevalence of tobacco use and its association with low BMI raises important questions about its impact on public health in India, a country which has a high prevalence of low BMI among adults. The focus of this paper is to provide a more detailed analysis of the relation between different forms of tobacco use and BMI and discuss the public health implications of these associations.

## Methods

The data presented in this report were obtained from a baseline cross-sectional survey conducted between 1992–1994 for a cohort study on tobacco-attributable mortality. [13] The survey was carried out in the main city of Mumbai, formerly Bombay – the largest city in India. A sampling frame was constructed from the electoral rolls. The sampling unit was a 'polling station,' consisting of 1000 to 1500 eligible voters. Rolls were assumed complete, as they are updated before every major election through house-to-house visits. Electoral rolls were organised by geographic areas. The selection of polling stations was done in a non-random manner to exclude those with apartments having high security, as it became evident during the pilot study that it would not be possible to gain access to these apartments.

Investigators approached all individuals aged ≥ 35 years (cut off chosen because of the overall goal of studying tobacco-attributable mortality in the cohort) listed in the selected polling stations for interview and anthropometric measurements. Individuals not present on the voters' list also were interviewed and included in the sample if their residence status was confirmed by their having a 'ration card.' These cards, issued by the Bombay Municipal Corporation, act as a proxy for residence cards and permit access to all city and state government services (including receiving certain food items at subsidized prices). Such individuals comprised about 5% of the sample. Less than 1% of individuals approached refused for interview and/or allow anthropometric measurements to be taken. A total of 99,958 adults, 40,071 males and 59,527 females, were recruited and surveyed. The study satisfied all criteria of ethical treatment of human subjects; especially those formulated by the Indian Council of Medical Research.

The survey included two components: 1. measurement of height, weight, blood pressure; and 2. interviewer-administration of a structured questionnaire to obtain information on age, occupational history, education, religion, language, and tobacco-related behaviour. Weight was measured using a bathroom scale accurate to 0.5 kg. The scale was kept on a flat surface and the subject was requested to step on it in bare feet without holding on to anything. Subjects were measured in normal apparel, which in Mumbai is light cotton because of the tropical weather year around. The weight was recorded to the nearest kg. Height was measured using a specially constructed instrument consisting of a steel platform to which was attached a steel measure tape. With the subject standing erect on the steel platform, the tape was pulled vertically above the head, and then brought down to touch the flat ruler placed horizontally on the crown of the head. Height was recorded to the nearest cm.

All data were entered directly in a handheld computer in the field and transferred to a PC once a week in the Project office. Respondents were classified according to present and past tobacco use as (a) having never used tobacco, (b) ex-smoker, (c) ex-smokeless tobacco use, (d) ever smoker, (f) ever smokeless tobacco user, and (g) ever mixed habit (smokeless and smoking). Ever smokers and ever mixed users clubbed together were further divided into those who smoked i) cigarettes ii) bidis and iii) cigarettes + bidis. The smokeless-tobacco use further divided into -i) mishri ii) mishri + other forms of tobacco iii) betel quid iv) other tobacco and v) areca nut. For analysis of educational background, respondents were classified as (a) illiterate - received no education (b) primary - up to five years of education (c) middle - 6 to 8 years of education (d) secondary - 9 to 12 years of education (e) college - those who have received education past secondary level. Religion and mother tongue was used for analysis of cultural background. For mother tongue, the categories used were- Marathi, Hindi, Gujarati, Tamil, Urdu and other. For religion, the respondents were classified as Hindu, Muslim, Christian, Jew, Buddhist, and other.

Descriptive statistics (mean, standard deviation, and proportion within categories) were calculated for the total survey population and by tobacco use for both men and women separately. Because the number of ex smokers and cigarette smokers among women was small, separate analyses were not conducted on these two categories.

Multivariable analysis was performed using logistic regression. The response variable, BMI, was converted into a dichotomous variable by using two cut points: 18.5 and 25.0 kg/m^2^. These are the conventional cut points indicating underweight and overweight, respectively. Three possible models corresponding to these two cut-off points were fit. Age (in 5-year age groups), education, mother tongue, religion and tobacco use were fit as independent variables in the final model. For dose response, the number of times (per day) tobacco was smoked, chewed or applied was refereed as frequency of habit per day and grouped as 1 to 5/day, 6 to 10/day and ≥ 11/day.

## Results

In general, non-users of tobacco had higher BMI values in this population (Table 1). In women, the few cigarette smokers represented (n = 17) had slightly higher BMI values and did never users. In both genders bidi smoking, appeared to be strongly associated with lower average BMI in this population. The difference between bidi smokers and those with never users of tobacco was more pronounced in women (2.8 BMI units lower than for subjects who did not use tobacco in any form) compared with men (2.2 BMI units lower than those with no habits). Often concern is directed at individuals who are at the extremes of the relative weight distribution.

Table 2 shows that the prevalence of low BMI (<18.5 kg/m^2^) was higher amongst all forms of tobacco users. Amongst smokers, the prevalence of low BMI was 2.5 times higher in bidi-smokers. However, the prevalence of underweight in cigarette smokers was not very different compared to non-tobacco users. Smokeless tobacco users, of all types had high prevalence of low BMI.

Table 3 gives the odds ratios for low BMI associated with tobacco habits controlling for age, education, mother tongue, and religion. All forms of tobacco use (except areca nut) were associated with higher risk of low BMI. Bidi-smoking was associated with the highest risk amongst all forms of tobacco use. In men, the odds ratio for low BMI associated with bidi smoking was about twice as large compared with cigarette smoking. For smokeless tobacco use, the risk was greater in women compared with men.

Table 4 shows a dose-response gradient in the tobacco use-BMI relationship. The dose-response was significant for smoking as well as smokeless tobacco use among men as well as women.

Table 5 explores the dose-response relationship further with low BMI divided into three categories: < 16.0; 16.0–17.0; 17.0–18.5 with frequency of smoking among men and smokeless tobacco use among women. A clear gradient in odds ratios is seen for almost every row and every column.

On comparing adjusted odds ratios with unadjusted odds ratios (data not shown), the adjusted odds ratios were always smaller indicating that controlled variables were confounders. The differences however, were not large; the highest reduction in odds ratio was from 2.79 to 2.19 for women with BMI < 16.0 and frequency of smokeless tobacco use > 10 per day; suggesting that there was little possibility of residual confounding affecting the results.

## Discussion

In India, nearly half of all rural adults and a quarter of urban adults have a low BMI (i.e., <18.5 kg/m^2^). [14]. Although, chronic energy deficiency due to inadequate diet may be the main factor placing the population at risk of low BMI, factors other than diet may play a significant role in explaining the low BMI within this population. These factors may act directly (by affecting appetite or other aspects of physiology) or indirectly (by decreasing the purchasing power for food). From a public health perspective, unmasking these non-dietary determinants of low BMI in the population will help in understanding the impact of such exposures. The present study has found that all forms of tobacco use are associated with low BMI independent of (i.e., after accounting for) age, education, mother tongue, and religion in this population. Further, there exists a dose-response gradient and the response at every dose was higher in women compared with men, a relationship observed in many tobacco-health disease analysis, including cancer related end points [15,16]. This finding raises important questions about the magnitude of the adverse impact of tobacco use on the health status of the population.

### Tobacco use among the socio-economically disadvantaged communities

Previously we have reported that illiteracy is an independent risk for low BMI in this population (OR = 6.52; 95% CI 5.38 to 7.89 for men and OR = 4.83; 95% CI 3.71 to 6.28 for women, respectively). [12] In Mumbai, the prevalence of tobacco use (especially bidi smoking and chewing) is inversely related to education (a good proxy for poverty in this population). [13] Bidi smoking is more common than cigarette smoking among the illiterate in Mumbai. This is true for all of the South Asia where the prevalence of bidi-smoking is reported to be 21–56% among men. [17]

In the present study, we found bidi- smoking was associated with the highest risk of low BMI (adjusted for education). All forms of tobacco produce free radicals that deplete antioxidants like Vitamin C, E and carotenoids and cause oxidative damage to DNA, proteins and lipids. [18-21] Concentrations of nicotine, tar and other toxic agents are higher in bidis than cigarettes and bidi smoking has a greater physiological and biochemical effect than cigarette smoking. [9,17]

Antioxidant-rich foods such as green-leafy vegetables and fruits that may help reduce the oxidative stress caused by tobacco [22] are usually lacking in the diet of the poor [23]. This makes them more vulnerable to tobacco-induced oxidative stress with more damaging effects than in a well-nourished population. [24] Our studies, conducted in three different parts of rural India, indicate relatively low intakes of antioxidant nutrient intake among smokers [25-27], calling attention to this as a widespread public health concern. In this population, infectious agents and pollution are the other environment factors that may play a role in this interaction. Tobacco use [28-30] and poor nutrition [31] impair the immune system. Hence, tobacco users are more susceptible to infectious agents. This has been demonstrated for the relationship between pulmonary tuberculosis (TB) and smoking. [32,11] Smoking has been associated with higher relative risk of TB mortality and prevalence of active TB. This was true in both rural (RR 4.2, 95% CI 3.7 to 4.8) and urban (RR 4.5, 95% CI 4.0 to 5.0) India. [11] The risk was higher for bidi-smoking, the predominant smoking habit in this population. Thus, there is strong evidence that tobacco use in this population contributes to an increased burden of infectious disease. On the other hand, infections will further increase oxidative stress in tobacco-users. Hence, the interactions between malnutrition, tobacco use and infections make this group more vulnerable to smoking-related mortality and morbidity.

Besides the direct physiological effect, tobacco use among the economically disadvantaged is known to reduce the resources available to purchase food, clothing, health, and education, all factors that contribute to poor nutritional status. [33] This explains why changes in the relationship between BMI and smoking change with the secular trend toward affluence [4].

### Tobacco use among women of reproductive age

Smoking is not yet very common among Indian women. However, smokeless tobacco use among women is high. In our survey population 59% of women used smokeless tobacco. This is similar to that reported in other South Asian female populations. Some 49% of the UK- Bangladeshi female population and 59% of rural Malaysian females use smokeless tobacco. [34,35] In addition to causing oral cancer, smokeless tobacco use may be associated with increased risk of osteoporosis [36] and breast cancer. [37] In this study, we found that smokeless tobacco use is associated with a greater risk of low BMI in women compared to men. A similar increased risk for cancer from smokeless tobacco use was reported in women compared with men. [38] This may be a result of gender differences in the biology and/or nutritional status. Although the findings of this paper have implications for women of all ages, low BMI in women of reproductive age in the developing countries is associated with poorer reproductive outcomes. [39]. Smokeless tobacco use is associated with lower birthweight [40-43] and decreases gestational age at birth [43] in India. A prospective study on maternal determinants of low birth weight in India found that intake of micronutrient-rich foods was an important limiting factor for fetal growth independent of maternal age, height, and weight. [44] Hence, depletion of antioxidant micronutrients by the toxic agents in smokeless tobacco may be playing a role the biology of fetal growth restriction by smokeless tobacco. India has the highest prevalence and largest absolute share of low birth weight in the world. [45] Therefore, it would be important to estimate the contribution of the singular and combined effect of tobacco use, low intake of micronutrient- rich foods, and low maternal BMI to the burden of low birth weight in India.

### Policy implications

There should be no question that a major emphasis of any competent public health programs should be on preventing youth from starting to use tobacco and in supporting tobacco cessation efforts among those already addicted. Also, it is known that tobacco effects dietary requirements. For example, in the UK, the recommendations for vitamin C intake for smokers is higher (80 mg/day) compared to non-smokers (40 mg/day). [46] Thus far, nutritional recommendations for the Indian population have not distinguished between tobacco nonusers, or users of tobacco in any form. [47] Doing so would help to highlight both the scientific issues and be socioeconomic implications of creating even more stringent requirements for more expensive food, such as thoses rich in antioxidants.

### Limitations

The results in this paper are from a cross-sectional study so all limitations of cross-sectional data apply to them. In cross-sectional studies, exposure and outcome are assessed at the same time point, and one may not be sure which one came before the other? In the present data set there does not appear any specific reason to suggest that those with low BMI were more prone to start using tobacco. Cross sectional study results may also be affected by differential mortality rates in different subgroups of exposure and outcome. In our study the highest mortality would be most probably in low BMI – tobacco user group and lowest in normal BMI – non tobacco user group. Both these groups contribute to the numerator of odd ratio so the bias if any, is not unidirectional. Another limitation may seem to be the fact that we have not focused on overweight (BMI >25). This is however deliberate; the inverse association between overweight and tobacco use is well established and the same was reported in current data set. [12] The overweight category (BMI > 25.0) was excluded from the referent category (18.5 – 25) to guard against artificial inflation of odds ratios. '

## Conclusion

The effects of tobacco use on the incidence of certain diseases, particularly cancers of the aerodigestive tract and urinary bladder are well documented. The findings of this study that all forms of tobacco use are associated with low BMI (a proxy for nutritional status) suggest a strong need for further research on as tobacco use may have even more far-reaching public health implications in India than previously thought. If the association is evaluated as causal then tobacco control research and intervention also will benefit other public health goals on improved nutritional status and consequential health benefits,. These results have potential to affect the population living in the developing world.

## Competing interests

The author(s) declare that they have no competing interests.

## Authors' contributions

MS Pednekar conceptualized and conducted all data analysis presented here and participated in preparation and finalisation of the article. PC Gupta, HC Shukla, and JR Hebert participated in all aspects of the conceptualization and preparation of the article. All authors read and approve the final manuscript.

## Pre-publication history

The pre-publication history for this paper can be accessed here:



## Figures and Tables

**Table 1:** Anthropometric values (mean ± SE of mean) by gender and tobacco use

	**Men**					Women*				
		
	***n***	***Age (years)***	***Height (cm)***	***Weight (kg)***	***BMI (kg/m^2^)***	***n***	***Age (years)***	***Height (cm)***	***Weight (kg)***	***BMI (kg/m^2^)***
	
**Never users**	10493	49.6 ± 0.12	161.5 ± 0.07	59.0 ± 0.11	22.6 ± 0.04	23965	46.3 ± 0.07	148.9 ± 0.04	52.5 ± 0.07	23.7 ± 0.03
Ex-users										
Ex-smoking	693	59.7 ± 0.48	161.8 ± 0.26	57.9 ± 0.44	22.0 ± 0.15	-	-	-	-	-
Ex-smokeless	824	58.4 ± 0.43	160.3 ± 0.25	57.2 ± 0.38	22.2 ± 0.13	1284	53.7 ± 0.34	147.9 ± 0.17	49.5 ± 0.30	22.6 ± 0.13
Ex-mixed	270	61.3 ± 0.73	160.3 ± 0.42	57.3 ± 0.74	22.2 ± 0.25	-	-	-	-	-
**Ever users**										
Smoking	6017	50.8 ± 0.15	161.3 ± 0.09	55.8 ± 0.15	21.4 ± 0.05	159	54.5 ± 1.03	150.5 ± 0.63	48.8 ± 0.85	21.5 ± 0.34
Smokeless	18365	50.1 ± 0.08	160.5 ± 0.05	56.1 ± 0.08	21.8 ± 0.03	35296	49.3 ± 0.06	147.3 ± 0.03	48.0 ± 0.06	22.1 ± 0.02
Mixed	5194	50.1 ± 0.16	161.6 ± 0.09	55.2 ± 0.15	21.1 ± 0.05	107	51.9 ± 1.19	150.0 ± 0.74	47.3 ± 1.09	21.0 ± 0.44
Types of smoking**										
Cigarette	4980	49.0 ± 0.16	162.6 ± 0.09	59.1 ± 0.16	22.3 ± 0.05	17	51.5 ± 2.20	157.6 ± 1.50	60.2 ± 1.48	24.3 ± 0.72
Bidi	5290	51.9 ± 0.16	160.2 ± 0.09	52.5 ± 0.14	20.4 ± 0.05	217	54.2 ± 0.90	149.7 ± 0.52	46.8 ± 0.73	20.9 ± 0.30
Cigarette + bidi	739	49.9 ± 0.44	162.2 ± 0.25	54.5 ± 0.40	20.7 ± 0.14	9	57.7 ± 3.86	153.0 ± 3.56	50.6 ± 2.43	21.7 ± 1.05
**Types of Smokeless**										
Mishri	2724	48.9 ± 0.21	159.9 ± 0.13	56.4 ± 0.20	22.0 ± 0.07	15899	46.0 ± 0.08	147.7 ± 0.05	48.8 ± 0.08	22.4 ± 0.04
Mishri + other	7426	50.8 ± 0.13	160.0 ± 0.08	55.0 ± 0.12	21.4 ± 0.04	13401	52.5 ± 0.10	146.6 ± 0.05	46.5 ± 0.09	21.6 ± 0.04
Betel quid	4639	50.3 ± 0.17	161.1 ± 0.10	57.5 ± 0.16	22.1 ± 0.06	3844	50.8 ± 0.18	148.0 ± 0.10	49.3 ± 0.18	22.5 ± 0.08
Other tobacco	3234	49.1 ± 0.20	160.9 ± 0.12	56.4 ± 0.19	21.7 ± 0.07	942	50.0 ± 0.40	147.8 ± 0.21	47.7 ± 0.36	21.8 ± 0.16
Areca nut	342	51.7 ± 0.68	161.5 ± 0.36	59.4 ± 0.60	22.7 ± 0.20	1210	51.5 ± 0.35	148.0 ± 0.18	50.5 ± 0.33	23.0 ± 0.14

*Due to very small numbers of female ex-smokers and with ex-mixed habit the data are not included

**Ever smokers and mixed users were clubbed together

**Table 2:** Prevalence (%) of low and normal BMI by tobacco use with 95% confidence intervals

**Tobacco use**	**Low BMI (<18.5 kg/m^2^)**		**Normal BMI (18.5–25.0 kg/m^2^)**	
	**Men (95% CI)**	Women* (95% CI)	**Men (95% CI)**	Women* (95% CI)

**Never users**	13.5 (12.8 to 14.2)	13.2 (12.8 to 13.6)	62.4 (61.5 to 63.3)	50.1 (49.5 to 50.7)
**Ex-users**				
Smoking	18.9 (16.0 to 21.8)	-	61.0 (57.4 to 64.6)	-
Smokeless	15.7 (13.2 to 18.2)	19.2 (17.0 to 21.4)	63.5 (60.2 to 66.8)	51.9 (49.2 to 54.6)
Mixed	17.8 (13.2 to 22.4)	-	56.3 (50.4 to 62.2)	-
**Ever users**				
Smoking	25.2 (24.1 to 26.3)	28.1 (21.1 to 35.1)	58.8 (57.6 to 60.0)	54.1 (46.4 to 61.8)
Smokeless	19.9 (19.3 to 20.5)	23.3 (22.9 to 23.7)	62.1 (61.4 to 62.8)	52.2 (51.7 to 52.7)
Mixed	26.4 (25.2 to 27.6)	35.1 (26.1 to 44.1)	59.5 (58.2 to 60.8)	47.9 (38.4 to 57.4)
Types of smoking**				
Cigarette	16.3 (15.3 to 17.3)	-	60.8 (59.4 to 62.2)	-
Bidi	32.1 (30.8 to 33.4)	32.7 (26.5 to 38.9)	57.9 (56.6 to 59.2)	50.7 (44.0 to 57.4)
Cigarette + bidi	32.6 (29.2 to 36.0)	-	55.1 (51.5 to 58.7)	-
**Types of Smokeless**				
Mishri	17.1 (15.7 to 18.5)	20.9 (20.3 to 21.5)	64.1 (62.3 to 65.9)	52.6 (51.8 to 53.4)
Mishri + other	21.5 (20.6 to 22.4)	26.6 (25.9 to 27.3)	63.1 (62.0 to 64.2)	52.3 (51.5 to 53.1)
Betel quid	17.7 (16.6 to 18.8)	21.4 (20.1 to 22.7)	61.0 (59.6 to 62.4)	50.9 (49.3 to 52.5)
Other tobacco	20.0 (18.6 to 21.4)	27.0 (24.2 to 29.8)	61.2 (59.5 to 62.9)	50.4 (47.2 to 53.6)
Areca nut	12.3 (8.8 to 15.8)	17.2 (15.1 to 19.3)	63.7 (58.6 to 68.8)	52.1 (49.3 to 54.9)

*Data for female ex-smokers and cigarette not analysed because of small numbers

**Ever smokers and mixed users were clubbed together

**Table 3:** Adjusted odds ratio for low BMI (< 18.5) by tobacco use* (Referent category was non-tobacco users with normal BMI, 18.5–25.0)

	**OR (95% CI) †**	
**Tobacco use**	***Men***	***Women***
**Never users**	1.0	1.0
**Ever users**		
Smoking	1.80 (1.65, 1.96)	1.59 (1.09, 2.32)
Smokeless	1.28 (1.19, 1.38)	1.50 (1.43, 1.59)
Mixed	1.83 (1.67, 2.00)	2.19 (1.40, 3.41)
Types of smoking**		
Cigarette	1.22 (1.11, 1.35)	-
Bidi	2.36 (2.16, 2.58)	2.01 (1.48, 2.74)
Cigarette + bidi	2.64 (2.22, 3.13)	-
**Types of Smokeless**		
Mishri	1.18 (1.05, 1.33)	1.41 (1.32, 1.50)
Mishri + other	1.38 (1.26, 1.51)	1.72 (1.61, 1.83)
Betel quid	1.23 (1.12, 1.36)	1.46 (1.33, 1.60)
Other tobacco	1.37 (1.23, 1.53)	1.80 (1.53, 2.11)
Areca nut	0.89 (0.64, 1.25)^NS^	1.18 (1.00, 1.40)^NS^

*Results are controlled for age, educational status, mother tongue and religion

†All ORs were significant at p < 0.0001 except the one marked ^NS^

**Those who smoked and used smokeless tobacco were counted as smokers

**Table 4:** Adjusted odds ratio* for low BMI (<18.5)by frequency of tobacco use (Non tobacco users with normal BMI 18.5–25.0 were referent category)

	**OR (95% CI)**^†^	
**Tobacco use**	***Men***	***Women***
	
Never users	1.0	1.0
**Frequency of use per day**		
Smoking		
1–5	1.31 (1.17 to 1.45)	1.50 (1.01 to 2.21)
6–10	1.81 (1.62 to 2.01)	2.02 (1.04 to 3.90)
≥ 11	2.11 (1.93 to 2.30)	2.20 (1.28 to 3.79)
Smokeless		
1–2	1.25 (1.13 to 1.38)	1.46 (1.38 to 1.55)
3–5	1.39 (1.28 to 1.52)	1.59 (1.50 to 1.69)
6–10	1.25 (1.14 to 1.38)	1.72 (1.58 to 1.88)
≥ 11	1.40 (1.20 to 1.62)	2.08 (1.77 to 2.45)

*Results are controlled for age and level of education

^† ^All ORs and trends were highly significant (p < 0.0001)

**Table 5:** Adjusted odds ratio* for three categories of low BMI and frequency of tobacco use(Non-tobacco users with normal BMI 18.5–25.0 were referent category)

**Frequency of tobacco use/day**			
**Smoking **(Men)	Mild thinness (BMI 17.0–18.5)	Moderate thinness (BMI 16.0–17.0)	Severe thinness (BMI<16.0)
1–5	1.21 (1.06 to 1.39)	1.37 (1.13 to 1.68)	1.45 (1.19 to 1.77)
6–10	1.68 (1.46 to 1.93)	1.93 (1.58 to 2.35)	1.97 (1.62 to 2.39)
> = 11	1.86 (1.66 to 2.09)	2.22 (1.88 to 2.62)	2.54 (2.17 to 2.98)
**Smokeless **(Women)			
1–2	1.32 (1.22 to 1.43)	1.50 (1.34 to 1.68)	1.72 (1.55 to 1.90)
3–5	1.46 (1.35 to 1.57)	1.71 (1.53 to 1.91)	1.76 (1.59 to 1.95)
6–10	1.59 (1.42 to 1.78)	1.63 (1.38 to 1.92)	2.05 (1.78 to 2.35)
> = 11	1.84 (1.48 to 2.29)	2.46 (1.87 to 3.24)	2.19 (1.70 to 2.84)

* Results are controlled for age and level of education
